# Population-based Laboratory Surveillance for AmpC β-Lactamase–producing *Escherichia coli,* Calgary

**DOI:** 10.3201/eid1303.060447

**Published:** 2007-03

**Authors:** Johann D.D. Pitout, Daniel B. Gregson, Deirdre L. Church, Kevin B. Laupland

**Affiliations:** * University of Calgary, Calgary, Alberta, Canada

**Keywords:** Surveillance, *E. coli*, AmpC β-lactamases, community-onset infections, research

## Abstract

In the Calgary Health Region during 2000–2003, prospective, active, population-based laboratory surveillance for all cefoxitin-resistant *Escherichia coli* isolates was performed. Isolates were screened with an inhibitor-based disk test, and plasmid-mediated types were identified by multiplex PCR with sequencing. A total of 369 AmpC β-lactamase–producing *E. coli* isolates were identified; annual incidence rates were 1.7, 4.3, 11.2, and 15 per 100,000 residents for each year, respectively. AmpC β-lactamase–producing *E. coli* was 5× more likely to be isolated from female than male patients across all age groups except <1 year. Of these isolates, 83% were community onset, and urine was the principal site of isolation (90% of patients). PCR showed that 125 (34%) were positive for *bla*_cmy_ genes; sequencing identified these enzymes to be CMY-2. In this large Canadian region, AmpC β-lactamase–producing *E. coli* is an emerging community pathogen that commonly causes urinary tract infections in older women.

## Abstract 2

AmpC β-lactamase–producing *E. coli* are commonly isolated from the urinary tract of older women.

Organisms that produce plasmid-mediated AmpC β-lactamases were first reported in the 1980s (*1*). These β-lactamases are derivatives of the chromosomally encoded clavulanate-resistant AmpC cephalosporinases of bacteria such as *Enterobacter* spp., *Citrobacter freundii, Morganella morganii, Aeromonas* spp., and *Hafnia alvei* (*2*). These enzymes have been reported in *Escherichia coli*, *Klebsiella pneumoniae*, *K. oxytoca*, *Salmonella* spp., *Enterobacter aerogenes*, and *Proteus mirabilis.* Because the genes are typically encoded on large plasmids that contain additional antimicrobial resistance genes, therapeutic options are limited (*3*).

*E. coli* possess a chromosomal gene that encodes for an AmpC β-lactamase. Usually, low amounts of β-lactamases are produced because the AmpC gene is regulated by a weak promoter and a strong attenuator. These *E. coli* isolates are sensitive to the cephamycins (*4*). However, surveys of resistance mechanisms in cephamycin-resistant isolates have identified promoter or attenuator mutations that result in the upregulation of AmpC β-lactamase production; these isolates are referred to as AmpC hyperproducers (*5*). Occasionally, cephamycin-resistant strains produce plasmid-mediated β-lactamases such as CMY-2, which are derived from bacteria with chromosomally encoded AmpC cephalosporinases (*3*). In addition, altered expression of outer membrane proteins constituting porins can also contribute to cephamycin resistance (*6*).

Methods for detecting *E. coli* AmpC hyperproducers or isolates that produce plasmid-mediated cephalosporinases are technically demanding for clinical laboratories. Although nonsusceptibility to the cephamycins suggests increased production of AmpC β-lactamases, organisms that produce these types of enzymes often go undetected and have been responsible for several nosocomial outbreaks (*2*,*7*).

Surveillance studies of organisms that produce plasmid-mediated AmpC β-lactamases, especially among community isolates, are needed (*8*). We noticed an increase in cephamycin-resistant *E. coli* isolates in the Calgary Health Region (CHR) during 2002 and 2003 (from 0.1% of all *E. coli* isolated in 2000 to 1.3% in 2003). To our knowledge, no surveillance studies have investigated the population-based epidemiology of AmpC β-lactamase–producing *E. coli* (i.e., hyperproducers or plasmid-mediated enzymes), although studies have shown the widespread distribution of these isolates in Europe and North America (*2*,*9*–*11*). Our objectives were to define the population-based incidence of infections caused by *E. coli* that produce increased levels of AmpC β-lactamases in a large well-defined Canadian region and investigate whether plasmid-mediated types were present in this population.

## Methods

### Patient Population

CHR provides all publicly funded healthcare services to the >1 million persons residing in the cities of Calgary and Airdrie and numerous adjacent surrounding communities covering an area of 37,000 km^2^ (www.health.gov.ab.ca/regions/RHA_comm3.html). Acute care is provided mainly through 1 pediatric hospital and 3 large hospitals for adults. A centralized laboratory (Calgary Laboratory Services; CLS) performs the routine clinical microbiology services for the community, e.g., nursing homes, physicians’ offices, community collection sites (where outpatients submit specimens for investigation purposes), and hospital sites within the CHR. Our base study population consisted of all patients from whom cefoxitin-resistant *E. coli* was first identified by CLS from January 1, 2000, through December 31, 2003.

### Population-based Surveillance.

Prospective, active, population-based, laboratory surveillance for all cefoxitin-resistant *E. coli* isolates was performed by CLS; all cefoxitin-resistant *E. coli* isolates were included in this study. We used the laboratory information system at CLS (PathNet Classic version 306, Cerner, Kansas City, MO, USA) to determine basic demographic information (age, sex, specimen submission site, date of hospital admission) and microbiologic data (location of isolate on patient and antimicrobial-susceptibility testing results) for all patients. Isolates were frozen and stored in batches for further analyses. Community-onset isolates were the first cultures obtained from outpatients or inpatients within 2 days of hospital admission. Hospital-onset isolates were the first cultures taken >2 days after admission to a hospital.

### Clinical Microbiology and Molecular Laboratory Testing

We included consecutive nonduplicate isolates of *E. coli* that were intermediately resistant or resistant to cefoxitin (MIC>8 μg/mL) and that were collected at CLS during January 2000 through December 2003 and isolated from clinical specimens by standard microbiology techniques. During June 2001, we cultured urine samples that had positive screening results from an ATPase-luciferase assay and those specifically requested by a physician (*12*). Strains were identified to the species level by using Vitek (Vitek AMS; bioMérieux Vitek Systems Inc., Hazelwood, MO, USA.). MICs to the following drugs were determined by Vitek: imipenem, gentamicin, tobramycin, trimethoprim-sulfamethoxazole, and ciprofloxacin. Results were interpreted according to the Clinical and Laboratory Standards Institute criteria for broth dilution (*13*).

Clinical isolates of cefoxitin-resistant *E. coli* were tested for AmpC β-lactamases by using the combination of the AmpC β-lactamase inhibitor Syn 2190 and cefotetan disks as described (*14*). All isolates with an AmpC β-lactamase were further investigated for plasmid-mediated AmpC β-lactamase genes by using multiplex PCR conditions and primers as described (*15*). These included enzymes that originated from the chromosomally encoded AmpC cephalosporinases of bacteria. Genes of the CMY-positive isolates were identified by cycle sequencing the full-length amplified products with conditions and different primers as described (*16*).

### Analysis

All analyses were performed by using Stata version 9.0 (Stata Corp., College Station, TX, USA). Variables were assessed before analysis by using histograms to identify underlying distribution. Means with standard deviations were used to describe normally or near normally distributed variables and were compared by using the Student *t* test. Medians with interquartile ranges (IQRs) were used to describe nonnormally distributed variables and were compared by using the Mann-Whitney U test. Differences in proportions were compared by using the Fisher exact test. Incidence rates (per 100,000 population per year) were calculated by using the annual number of new cases among CHR residents as the numerator and regional population estimates for each year from 2000 through 2003 (December 2003 boundaries) of the CHR as the denominator. Patients with Alberta healthcare numbers were considered CHR residents and were included; those with out-of-province healthcare numbers were excluded. Age- and sex-specific incidence rates were calculated by dividing the number of new cases within a subgroup by the population at risk. Risk ratios (RR) for incidence rates among demographic subgroups were calculated by dividing the incidence rate with the factor (as opposed to without) and were reported with 95% confidence intervals (CIs) as described (*17*).

## Results

During the 4-year study period, 78,275 *E. coli* isolates were obtained from 51,735 patients; 72,756 (93%) isolates were classified as community onset and 5,519 (7%) as hospital onset. For 408 (0.7%) patients, a cefoxitin-resistant *E. coli* isolate was identified. The number of isolates (per first isolate per patient per year) increased significantly each year: 23 (0.1%) of 17,989 patients in 2000, 53 (0.3%) of 15,907 in 2001, 141 (1%) of 14,583 in 2002, and 191 (1.3%) of 14,319 in 2003 (p<0.0001 for 2000 compared with 2003). Of the 408 cefoxitin-resistant isolates, 384 (94%) were available for further analysis. Of these, 369 (96%) were positive for AmpC β-lactamases according to the Syn 2190 inhibitor disk screen test, and of the 369, 359 (97%) were from CHR residents. The number of isolates identified in CHR residents during 2000, 2001, 2002, and 2003 were 18, 46, 123, and 172, with annualized incidence rates of 1.7, 4.3, 11.2, and 15 per 100,000, respectively.

**Figure 1:**
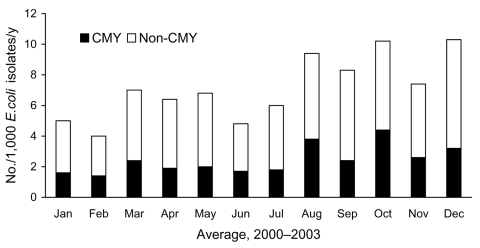
AmpC β-lactamase–producing *Escherichia coli* isolates per 1,000 *E. coli* isolates, Calgary Health Region, 2000–2003. Data are averaged over the 4-year period. The presence of plasmid-mediated AmpC β-lactamase genes was determined using multiplex PCR conditions and primers as described [*8*]. CMY; isolates positive for chromosomal gene of *Citrobacter freundii;* non-CMY; isolates negative by multiplex PCR.

**Figure 2:**
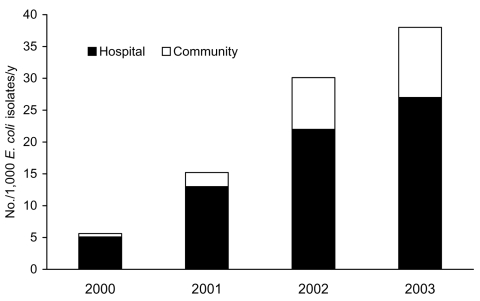
First AmpC β-lactamase–producing *Escherichia coli* isolates per 1,000 *E. coli* isolates per year. Calgary Health Region, 2000–2003. Community isolates were those obtained from outpatients or admitted patients who had their first cultures obtained within 2 days of hospital admission. First cultures from other hospitalized patients obtained after 2 days of admission were deemed to represent hospital-onset isolates.

Seasonal variability in the occurrence of AmpC β-lactamase–producing *E. coli* isolates was moderate; the lowest rates of isolation were in early winter and spring, and the highest rates were in late summer and fall (Figure 1). Of the 369 isolates, 61 (17%) were classified as hospital onset. Of the 308 (83%) community-onset isolates, 54 were submitted from hospital emergency departments, 24 from inpatients within the first 2 days of admission, 20 from nursing home residents, and the rest from outpatients. While the number of hospital-onset AmpC β-lactamase–producing *E. coli* isolates increased gradually during 2000–2003 (5, 12, 20, 24 each year, respectively), the number of community-onset isolates increased dramatically (13, 35, 108, 152 each year, respectively). The increase among the acute care centers was not the result of clustering of patients in a specific acute care center. However, in relation to all first *E. coli* isolates per year per patient tested by CLS, AmpC β-lactamase–producing *E. coli* were proportionally more likely to be obtained as hospital-onset isolates (Figure 2).

**Figure 3:**
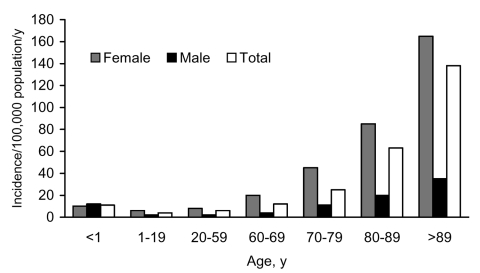
Age- and sex-specific incidence of AmpC β-lactamase–producing *Escherichia coli* isolates per 100,000 population, Calgary Health Region, 2000–2003.

The median age of the cohort was 51.1 (IQR 27.3–74.3) years; most (310; 84%) patients were female. Incidence of AmpC β-lactamase–producing *E. coli* significantly increased in association with increasing age (Figure 3). Risk for isolation of AmpC β-lactamase–producing *E. coli* was 5× higher for female than male residents (14.0 vs. 2.6 per 100,000 per year; RR 5.4; 95% CI 4.04–7.31; p<0.0001); this increased risk was observed across all age groups with the exception of the very young (< 1 year) (Figure 3).

Among the 369 AmpC β-lactamase–producing isolates, the principal site of isolation was the urinary tract for 333 (90%) patients, bloodstream for 20 (5%), respiratory tract for 8 (2%), soft tissue for 5 (1%), and abdomen for 3 (1%). Of these same 369 isolates, 73 (20%) were not susceptible to trimethoprim-sulfamethoxazole, 32 (9%) to tobramycin, 54 (14%) to gentamicin, and 33 (9%) to ciprofloxacin. No resistance to imipenem was detected.

Multiplex PCR amplified a 462-bp amplicon among 125 (34%) of the 369 AmpC β-lactamase–producing isolates that was consistent with the plasmid-encoded types of AmpC β-lactamases originating from the chromosomal gene of *Citrobacter freundii* (CMY types) (*15*). No other types of plasmid-mediated AmpC enzymes were present. Sequence analysis of full-length PCR products on 15 randomly selected isolates showed 100% identity to *bla_CMY-2_* (*18*). The Table shows features of CMY-type and non–CMY-type AmpC β-lactamases. With the exception of a higher rate of gentamicin resistance among CMY-2–positive strains (Table), *E. coli* isolates that produced CMY-types and those that produced non-CMY types of AmpC β-lactamases did not differ according to year of study, principal site of isolation, or demographics.

**Table:** Features of *Escherichia coli* β-lactamases isolated from Calgary Health Region, 2000–2003

Feature*	CMY types (n = 125), no. (%)	Non-CMY types (n = 244), no. (%)	p value
Female	105 (84)	205 (84)	1.0
Community-onset	104 (83)	204 (84)	1.0
Resistant to			
Ciprofloxacin	10 (8)	23 (9)	0.6
Gentamicin	28 (22)	26 (11)	0.004
Tobramycin	13 (10)	19 (8)	0.4
Trimethoprim-sulfamethoxazole	29 (23)	44 (18)	0.3

*Median age for those with CMY types was 54.3 (interquartile range 27.7–76.1); median age for those with non-CMY types was 50.5 (interquartile range 26.6–73.3).

## Conclusion

Limited data are available regarding hyperproduction of AmpC β-lactamases among *E. coli* in the United States and Canada. A study from Canada showed that most cephamycin-resistant *E. coli* from the Toronto area in 2001 had different promoter and attenuator mutations in the chromosomal AmpC cephalosporinases (*5*). Jacoby and colleagues found plasmid-mediated AmpC-type resistance in 7 of 75 of ceftazidime-resistant *E. coli* from 25 US states; 2 of these isolates produced CMY-2 (*19*). Mulvey and colleagues studied 232 cefoxitin-resistant *E. coli* from 12 hospitals in Canada and found that 25 (11%) strains contained CMY-2 and 51 (22%) had different promoter and attenuator mutations (*20*). *E. coli* that produce CMY-2 have also had been isolated from food-producing animals in Canada and the United States (*21*,*22*).

Laboratory tests that use inhibitors of AmpC β-lactamases in *E. coli* successfully distinguish between isolates that have altered expression of outer membrane proteins and isolates that produce increased levels of AmpC β-lactamases (*23*,*24*). Multiplex PCR that detects the different types of plasmid-mediated AmpC β-lactamases is the most practical way to differentiate between isolates with promoter or attenuator mutations and those with plasmid-mediated cephalosporinases (*15*). Our study screened all cefoxitin-resistant *E. coli* for AmpC β-lactamases and used multiplex PCR to identify plasmid-mediated types. However, the cephalosporinases that originated from *H. alvei* (e.g., ACC types) are not detected by our phenotypic method. In our study, >90% of cefoxitin-resistant strains produced increased levels of AmpC cephalosporinases; 125 (34%) of these 369 were positive for CMY-2, much higher than the 11% reported by Mulvey et al. (*20*). None of the other plasmid-mediated AmpC types were present in *E. coli* isolated from patients in the CHR.

Some studies have recognized a role of AmpC β-lactamase–producing *E. coli* in nosocomial infections (*2*,*3*,*7*); however, these studies were based at institutions and did not survey community-based laboratories. Because our surveillance included all clinical specimens from hospital and community sites, we are highly unlikely to have missed many isolates. We observed that in the CHR, AmpC β-lactamase–producing *E. coli* is predominantly a community-onset pathogen. The designs of other studies in the literature (*19*,*20*) make it unclear whether AmpC β-lactamase–producing *E. coli* is an important cause of community-onset infections elsewhere. A community outbreak in CHR during 2002 resulted from CMY-2–producing *Salmonella enterica* serotype Newport associated with the handling of pet treats (*25*). A previous study from our center has shown that these salmonella isolates share similar-size plasmids with CMY-2–producing *E. coli* of multiple pulsed-field gel electrophoresis types identified in our study (D.B. Gregson, unpub. data). Thus, *E. coli* and *Salmonella* spp. may share similar plasmids.

Several investigations have shown that animals may represent a source for dissemination of AmpC-encoding genes from *E. coli* to humans. Evidence of CMY-2–producing isolates in cattle (*26*), pork (*27*), poultry (*21*,*28*), and dogs and cats (*29*) is of concern because food-producing animals and domestic pets may act as reservoirs for resistant organisms. Therefore, factors that lead to the high rate of isolation of AmpC β-lactamase–producing *E. coli* in patients from the community require further exploration.

Ours is the first report of the population epidemiology of AmpC β-lactamase–producing *E. coli*. We restricted our study to *E. coli* because other plasmid-mediated AmpC-producing organisms are rare in our region (only 17 patients infected with AmpC-producing *K. pneumoniae* and 12 with AmpC-producing *Salmonella* spp. were identified at CLS during this study period). We determined demographic risk factors associated with the isolation of AmpC β-lactamase–producing *E. coli* by comparing patient demographic characteristics with our well-defined base population. In these analyses, female and older patients were at much higher risk than male and younger patients (Figure 3). These results are similar to those we obtained in a previous study from CHR that investigated the population epidemiology of infections caused by extended-spectrum β-lactamase (ESBL)–producing *E. coli* during 2000–2002 (*30*). Incidence of ESBL-producing isolates was stable for the 3 years; incidence rates in 2000, 2001, and 2002 were 5.0, 5.6, and 5.7 per 100,000, respectively. These rates differ from those for AmpC β-lactamase–producing *E. coli* in this study, which had rates of 1.7, 4.3, and 11.2 per 100,000 for these 3 years, respectively. Our previous study also showed that most ESBL-producing *E. coli* from our region isolated during the same period were resistant to gentamicin and ciprofloxacin (*30*); in our current study, only 14% and 9% of AmpC β-lactamase–producing isolates were resistant to gentamicin and ciprofloxacin, respectively. Thus, susceptibility patterns differ between ESBL- and AmpC β-lactamase–producing *E. coli* from the CHR isolates during the same period.

The population-based design has some methodologic limitations. First, because this was a laboratory-based study, detailed clinical information (e.g., prior receipt of antimicrobial drugs, travel, exposure to food and water, underlying concurrent conditions) was not available. We were therefore unable to determine whether the isolates in this study truly caused infection. The lack of detailed clinical information is an inherent limitation to all laboratory-based studies. Second, isolates were defined as either community- or hospital-onset on the basis of their location of submission. Although this may in part reflect where these organisms were acquired, some isolates classified as community onset may have been associated with healthcare (*31*). Third, incidence rates were based on the assumption that all persons with Alberta healthcare numbers were CHR residents. We estimate that 10%–15% of patients in this study may have resided within other health regions in Alberta rather than within CHR. As a result, our incidence rates may be slightly higher than the true values.

In conclusion, this study demonstrates that AmpC β-lactamase–hyperproducing *E. coli* is an emerging community pathogen in the CHR with public health implications. Our results warrant increased efforts at surveillance for and the study of risk factors associated with the acquisition of these isolates in order to guide future prevention and control measures
